# Supplementary material for: Achieving tissue-level softness on stretchable electronics through a generalizable soft interlayer design
Source: Nat Commun. 2023 Jul 26;14:4488. doi: 10.1038/s41467-023-40191-3 (PMC10372055; doi:10.1038/s41467-023-40191-3)
Supplement: Supplementary file 1 — Supplementary information [file 41467_2023_40191_MOESM1_ESM.pdf]

Supplementary Information

Achieving tissue-level softness on stretchable electronics through a generalizable soft interlayer design

Yang Li[1], Nan Li[1], Wei Liu[1], Aleksander Prominski[2], Seounghun Kang[1], Yahao Dai[1], Youdi Liu[1], Huawei Hu[1], Shinya Wai[1], Shilei Dai[1], Zhe Cheng[2], Qi Su[1], Ping Cheng[1], Chen Wei[3], Lihua Jin[3], Jeffrey A. Hubbell[1], Bozhi Tian[2], Sihong Wang[1,4,*]

[1]Pritzker School of Molecular Engineering, The University of Chicago, Chicago, IL, 60637, USA

[2]Department of Chemistry, The University of Chicago, Chicago, IL, 60637, USA

[3]Department of Mechanical and Aerospace Engineering, University of California, Los Angeles, Los Angeles, CA, 90095, USA

[4]Nanoscience and Technology Division and Center for Molecular Engineering, Argonne National Laboratory, Lemont, IL, 60439, USA

Email: sihongwang@uchicago.edu

Material synthesis

Synthesis procedure of DPPT-TT

A mixture of DPP (111 mg, 0.109 mmol, 1.0 eq.), 2,5-bis(trimethylstannyl)-thieno[3,2-b]thiophene (51 mg, 0.109 mmol, 1.0 eq.), $Pd_2(dba)_3$ $CHCl_3$ (2.2 mg, 0.0022 mmol, 0.02 eq.), and $P(o\text{-tol})_3$ (2.6 mg, 0.0087 mmol, 0.08 eq.) was added 2 mL of chlorobenzene in a nitrogen filled glovebox. The reaction vial was sealed and submitted to a microwave reactor with the following temperature profile: 1 minute at 100 °C, 2 minutes at 120 °C, 2 minutes at 140 °C, 3 minutes at 160 °C and 40 minutes at 180 °C. After the reaction was cooled down, 10 mol% of trimethyl(phenyl)stannane were added and the crude polymer solution was heated again to 1 minute at 100 °C, 1 minute at 120 °C, 2 minutes at 140 °C and 3 minutes at 160 °C. To complete the end-capping of the polymer, 10 mol% of bromobenzene were added and the reaction vessel submitted one last time to microwave heating (1 minute at 100 °C, 1 minute at 120 °C, 2 minutes at 140 °C and 3 minutes at 160 °C). The crude polymer was then precipitated into methanol, filtered, loaded to a Soxhlet thimble and washed successively with hexane, acetone (each for 24 h). The polymer was finally collected from the thimble with chloroform. The chloroform solution was then concentrated and precipitated into methanol. The polymer fibers were collected by filtration and dried under high vacuum for 24 h to give the final polymers.

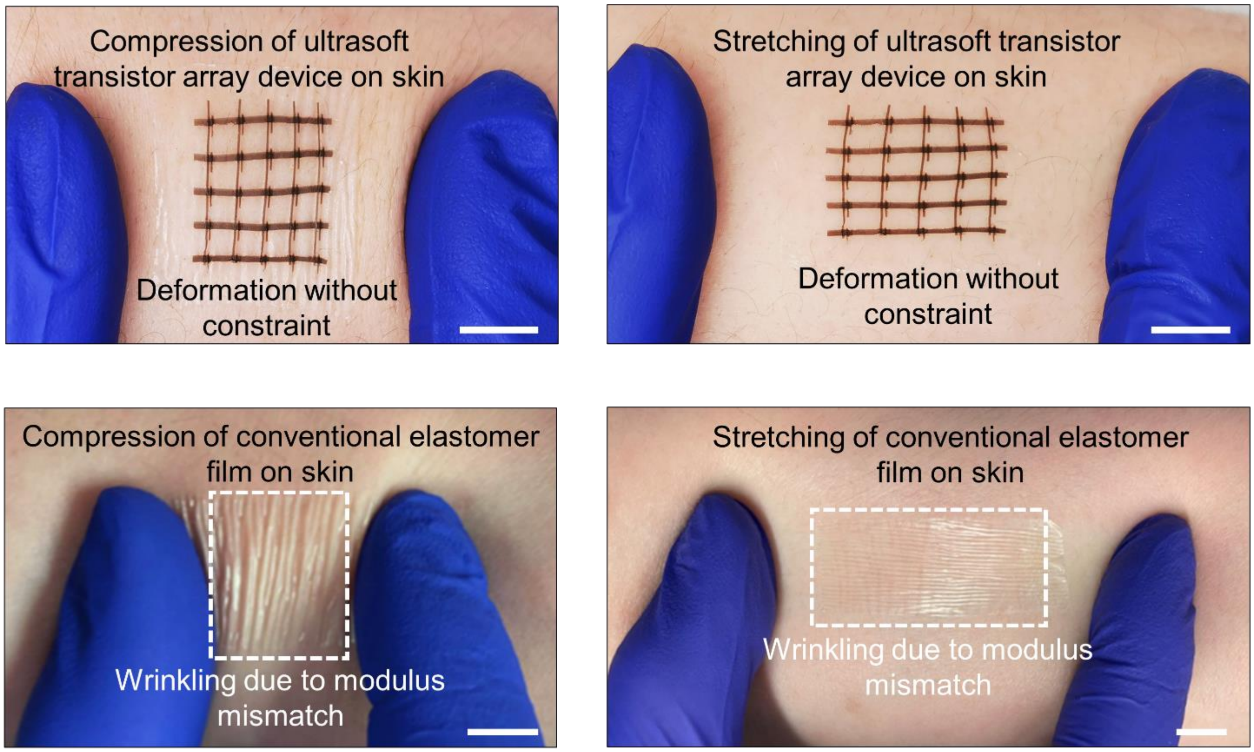

Supplementary Fig. 1. Compression and stretching of an ultrasoft transistor array device mounted on skin, and no constraint deformation is observed (top figure). When an MPa-level conventional elastomer thin film (i.e., 3M Tegaderm with a modulus of 7.4 MPa is used here) in a few MPa range is integrated on the skin, excessive wrinkles will form when the skin is under tensile or compressive strain (scale bars, 10 mm).

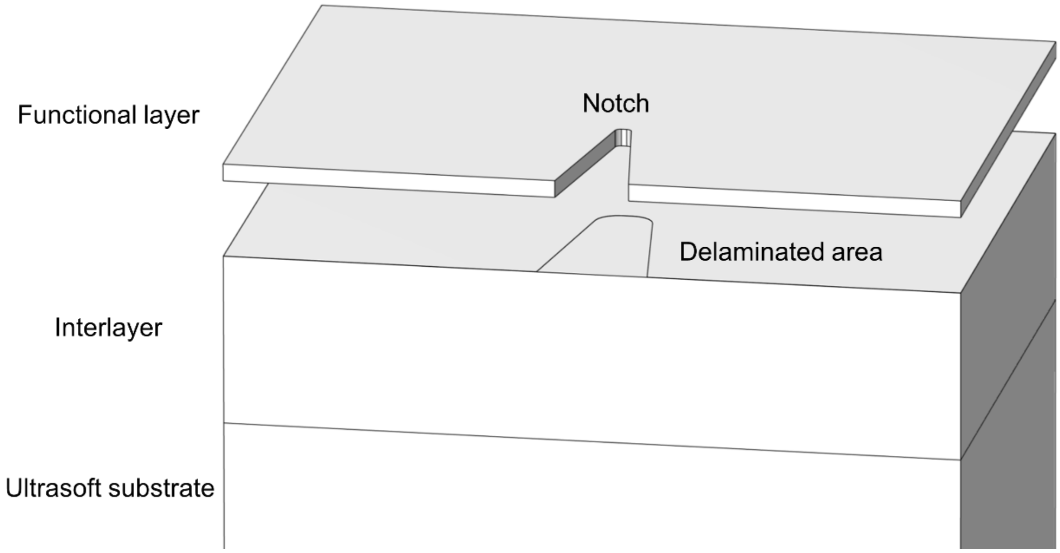

Supplementary Fig. 2. Simulation model for the delaminated notch. Showed in the schematics, the notch is opened in the functional layer, and a delaminated area is created by removing 1 nm thin layer in that area from the top surface of interlayer. Hence, there is no connection between the interlayer and the region near the notch. By increasing the dimension of delaminated area, stress level around the notch tip will be increased.

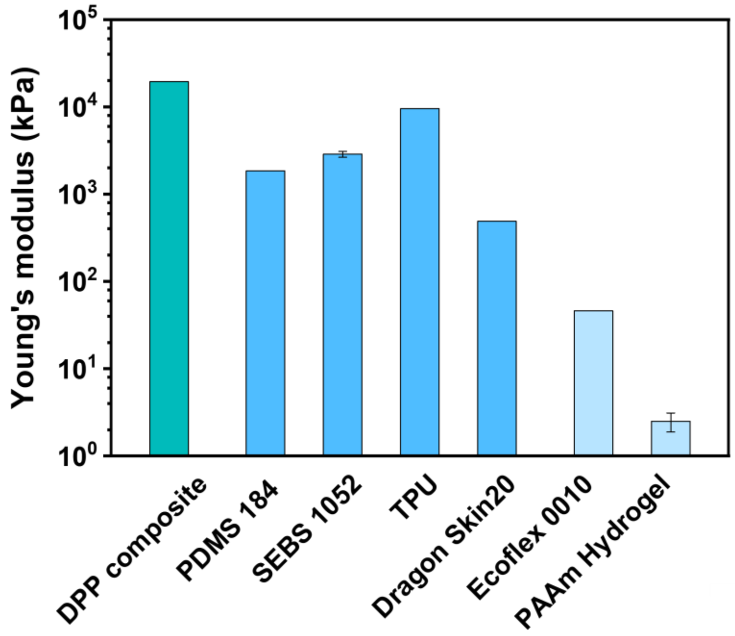

Supplementary Fig. 3. Young's moduli of stretchable materials. Nanoconfined semiconductor, 19.4 MPa; Sylgard 184 (PDMS, crosslinker: monomer ratio, 1:10) 1.85 MPa; SEBS H1052, 2.83 MPa; TPU, 9.56 MPa; Dragon Skin 20, 489 kPa; Ecoflex 0010, 46 kPa; ultrasoft PAAm hydrogel, 2.58 kPa. Modulus for Sylgard 184, Eco-Flex 0010, Dragon Skin, and thermal plastic polyurethane (Dow) are obtained from their datasheets. Modulus of DPP composite is cited from previously published report. Modulus of SEBS 1052 and PAAm hydrogel are measured using tensile tests.

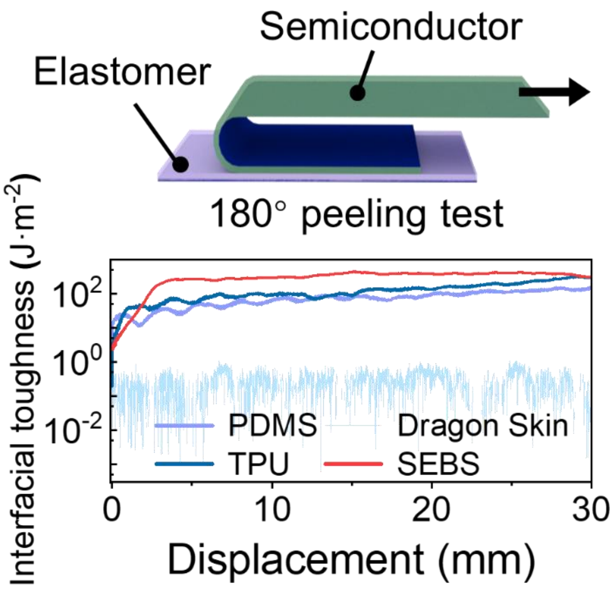

Supplementary Fig. 4. Peeling test for the polymer semiconductor on different elastomer substrates. The top schematic shows the 180° peeling process of a semiconductor layer from an elastomer substrate. Bottom figure shows the interfacial toughness between the semiconductor layer and different elastomers (PDMS, Dragon Skin, TPU and SEBS). Among them, SEBS provides the strongest adhesion with the semiconductor film.

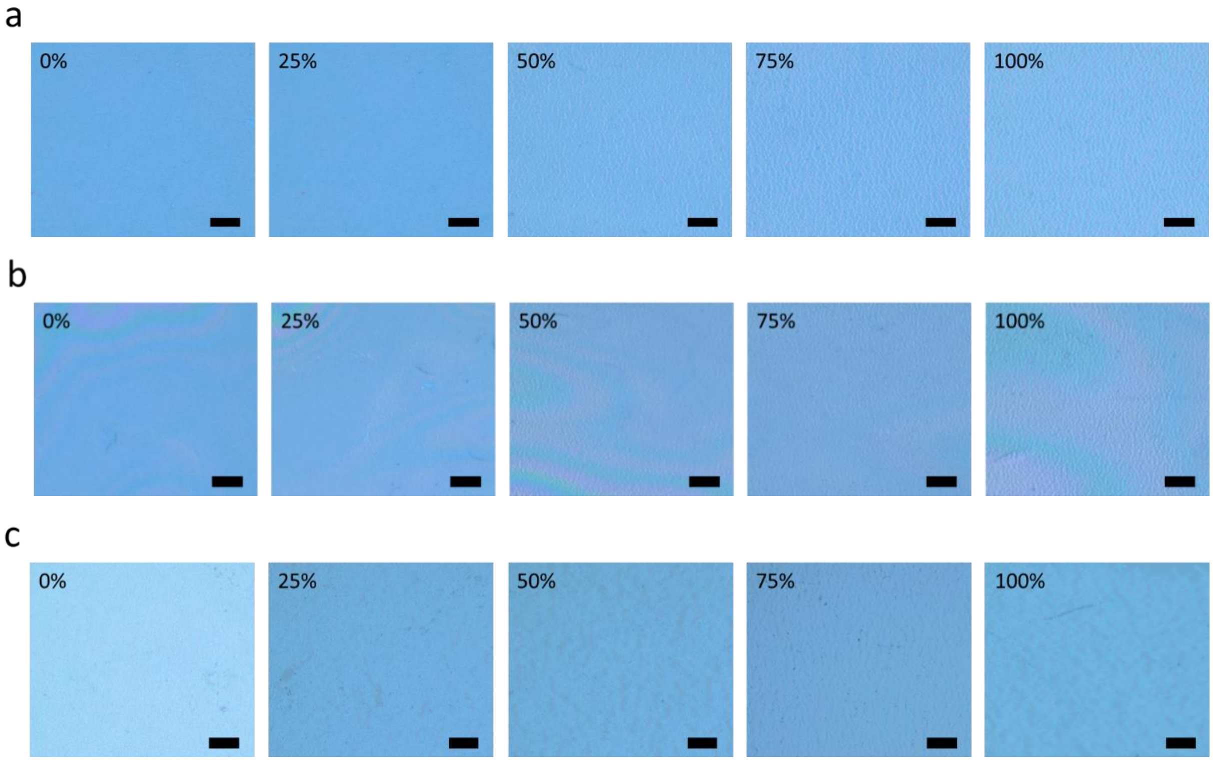

Supplementary Fig. 5. Representative optical images for cracks in PSC films supported by interlayers made of a, SEBS, b, TPU and c, PDMS. SEBS and TPU interlayers have similar mechanical buffering effects for delayed crack propagation in PSC films. For the PDMS interlayer, obvious cracks formed under 25% strain, and cracks at much larger scale are observed under 100% strain than the SEBS and TPU interlayers. Scale bars, 20 μm.

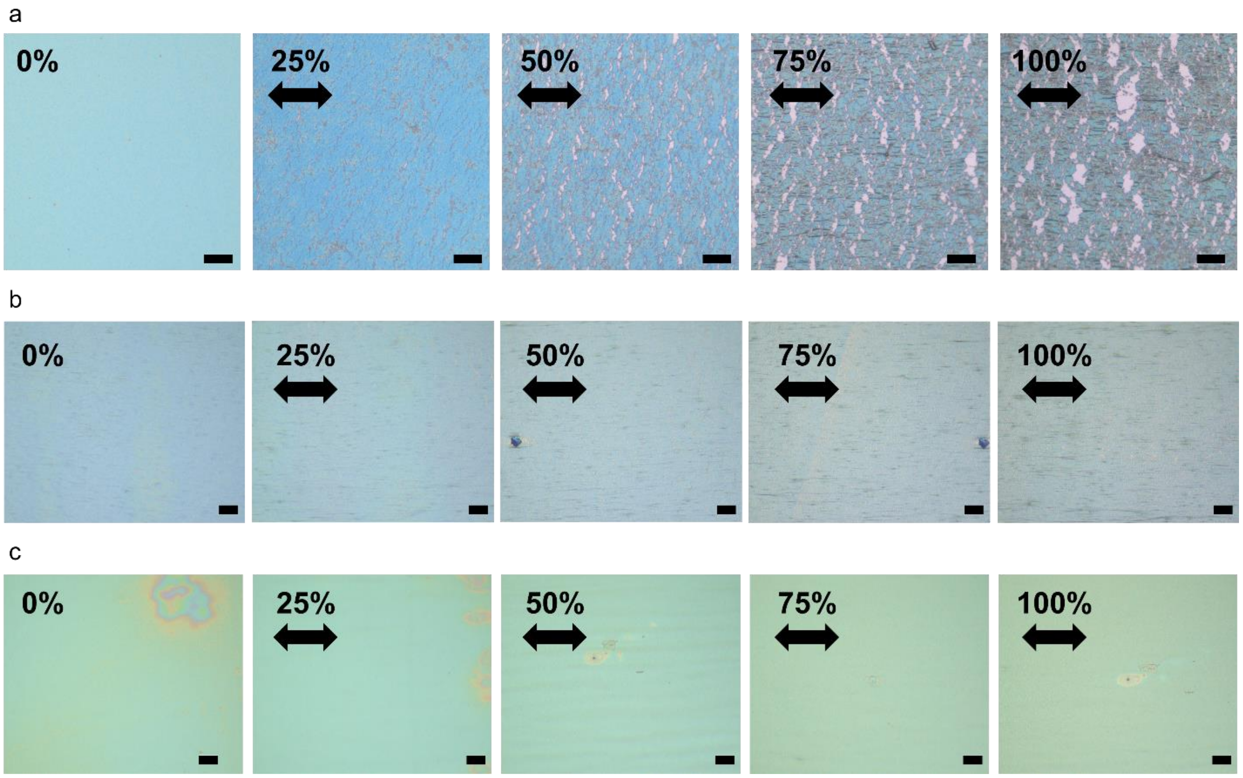

Supplementary Fig. 6. Representative morphology evolvement of the stretchable semiconductor film under stretching on three different types of substrates: a, On the Ecoflex substrate. **b**, On the Ecoflex substrate with a SEBS soft interlayer with a thickness of 200 nm. **c**, On the Ecoflex substrate with a SEBS soft interlayer with a thickness of 2 µm. Scale bars, 50 µm.

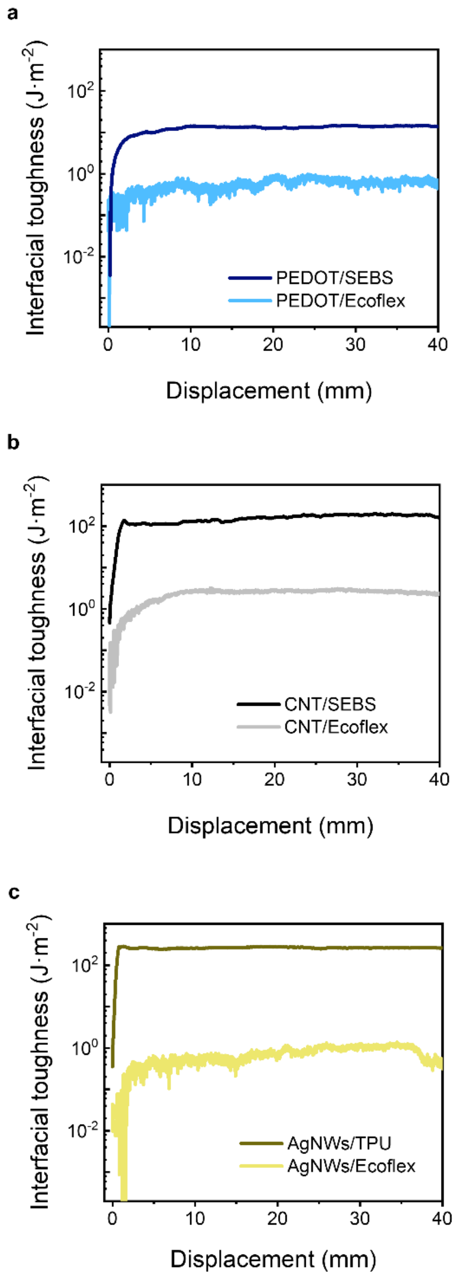

Supplementary Fig. 7. Interfacial toughness measurement for the stretchable conductors on ultrasoft silicone and interlayers. a, Interfacial toughness of PEDOT:PSS stretchable polymer conductor on SEBS and Ecoflex-0010. **b,** Interfacial toughness of CNT stretchable network on SEBS and Ecoflex-0010. **c**, Interfacial toughness of AgNWs stretchable network on thermal plastic polyurethane and Ecoflex-0010.

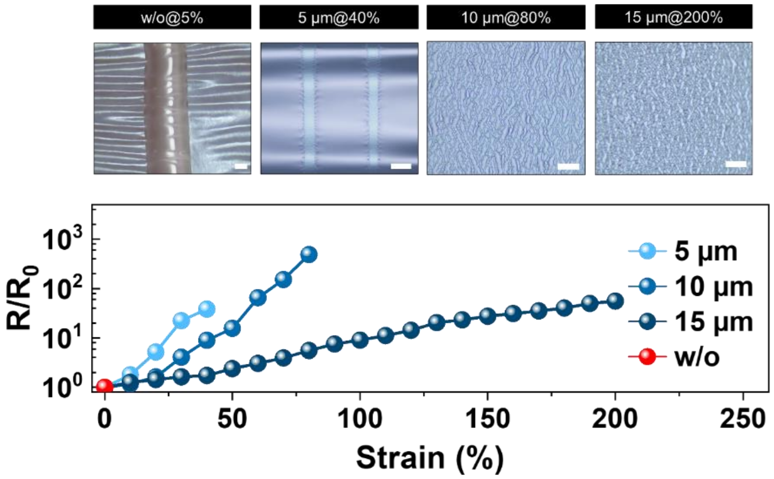

Supplementary Fig. 8. Morphology and electrical performance of PEDOT conductive composite thin film supported on interlayers with different thicknesses. Representative optical images for the PEDOT:PSS composite stretching on Ecoflex substrate with different thicknesses of SEBS interlayers (w/o, 5 µm, 10 µm, and 15 µm). The top figure shows the disconnecting strains of different interlayer thicknesses, and the bottom figure shows the resistance change with the increase of the strain. Scale bar, for w/o and 5 µm samples, 500 µm; for 10 µm and 15 µm samples, 100 µm.

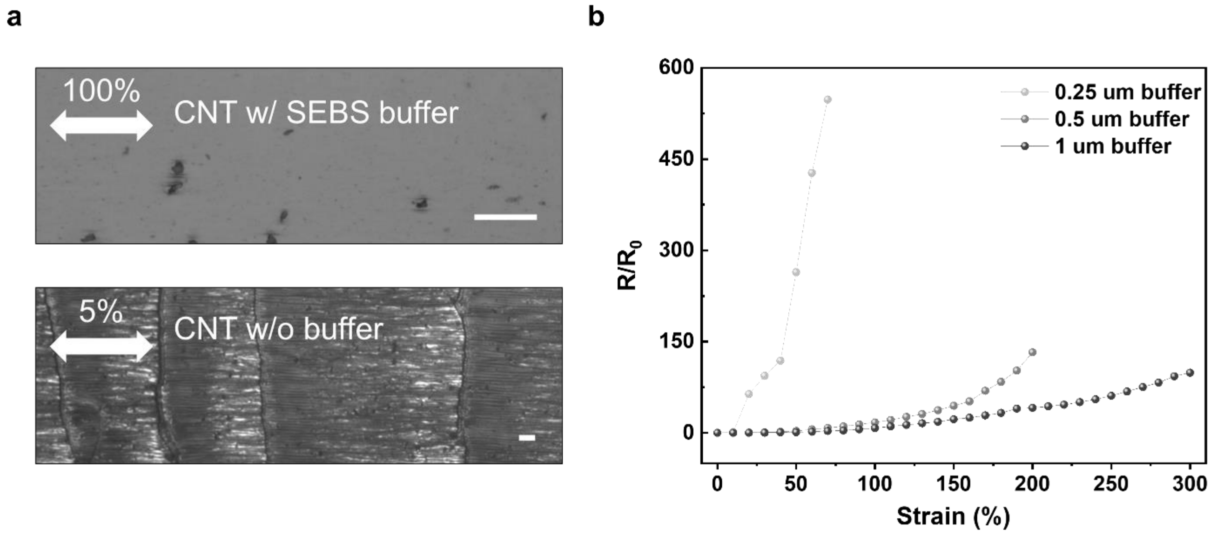

Supplementary Fig. 9. Stretching-induced morphology and resistance changes of CNT networks on Ecoflex substrates, with and without a soft interlayer. a, Representative optical microscopy images of CNT-based stretchable electrodes. The upper figure shows the buffered CNT electrode on 1-μm, SEBS, which does not form any cracks under 100% strain. The lower image shows the disconnected electrode without a soft interlayer, under only 5% strain (scale bar, 500 μm). **b**, Resistance measurement for the CNT electrodes under strain with different interlayer thicknesses.

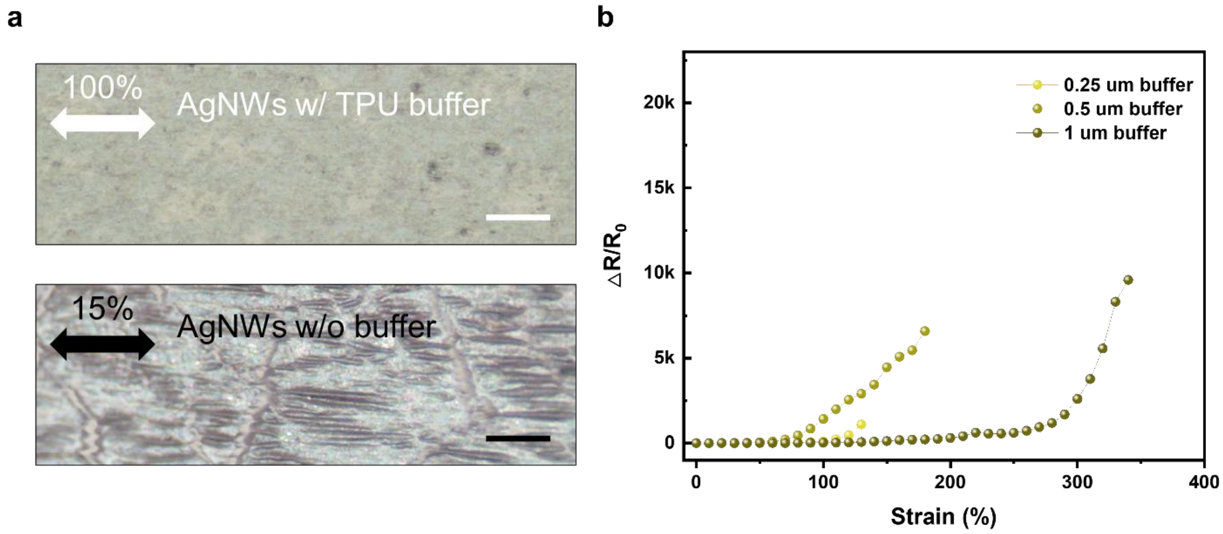

Supplementary Fig. 10. Morphology and electrical performance of AgNWs with different interlayer thicknesses. a, Representative optical microscopy for the silver nanowires based stretchable electrodes. The upper figure shows the buffered AgNWs electrode under 100% uniaxial strain without any cracks (1 μm, TPU based interlayer); the lower image shows the disconnected electrode without a soft interlayer, under only 15% strain. TPU is selected as the interlayer because of the intermediate Young's modulus between the soft substrate and the AgNWs layer and the high interfacial toughness (scale bar 500 μm). **b**, Resistance measurement for the AgNWs electrodes under strain with different interlayer thicknesses.

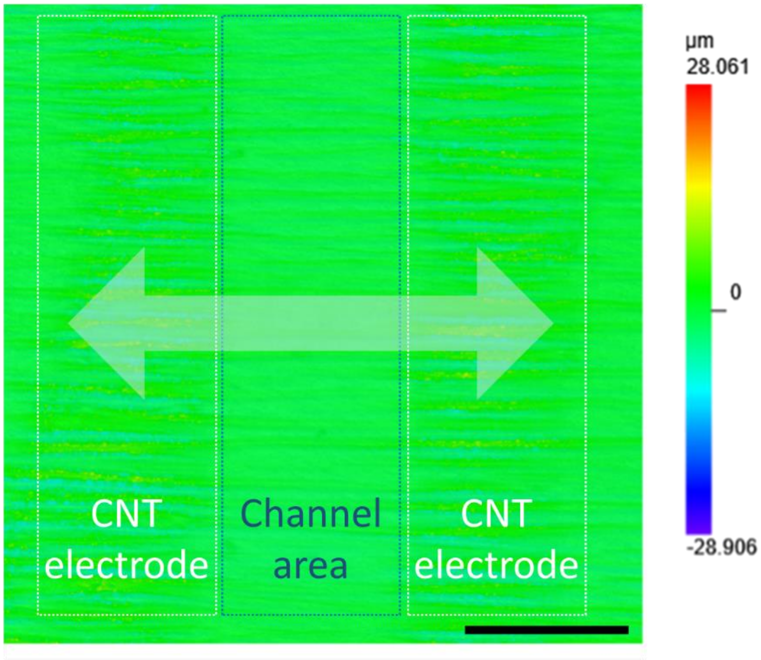

Supplementary Fig. 11. Confocal microscope imaging of a typical strained ultrasoft transistor device. Wrinkled structures are observed on the surface due to the modulus mismatch between functional layers and the ultrasoft hydrogel substrate (scale bar, 500 μm).

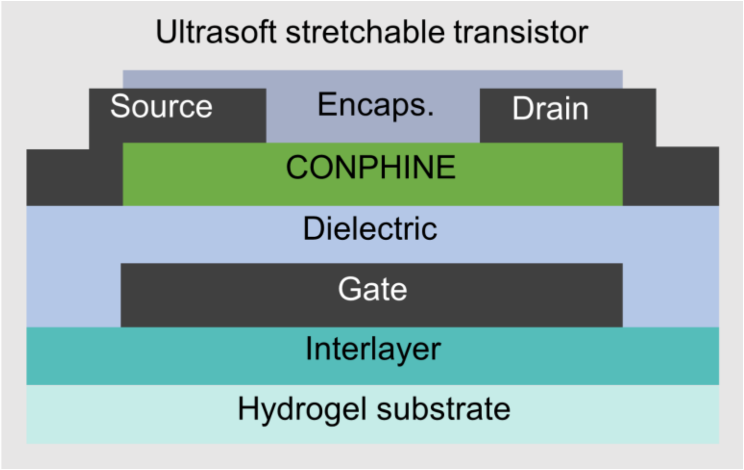

Supplementary Fig. 12. Schematic of the cross-section of an ultrasoft intrinsically stretchable transistor. Top encapsulation layer, SEBS H1052, 1.2 µm; semiconducting film, COHPHINE (blending DPPT-TT with SEBS H1221 with the ratio of 3:7), ~100 nm; dielectric layer, SEBS H1052, 1.8 µm; soft interlayer, SEBS H1052, 1.2 µm; PAAm hydrogel substrate, 200 µm.

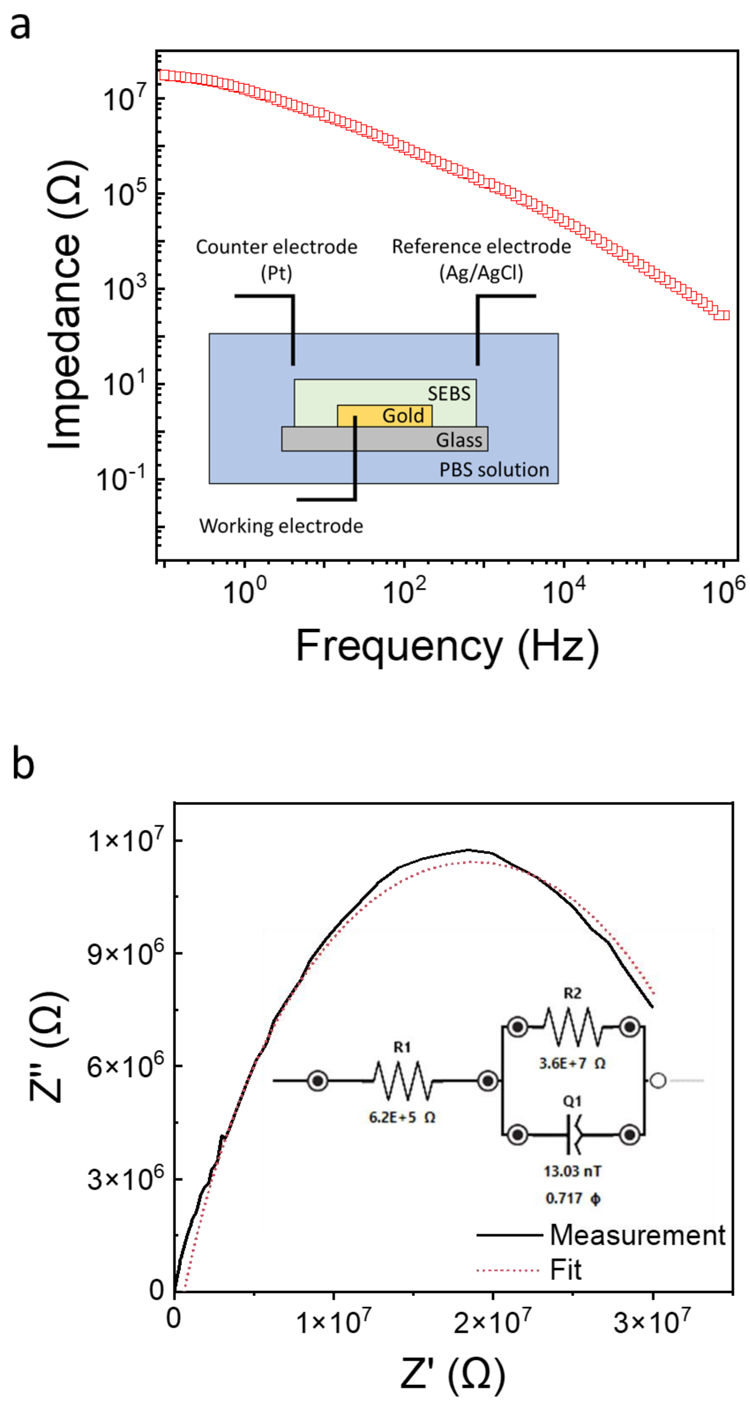

Supplementary Fig. 13. a, Electrochemical impedance spectroscopy (EIS) of SEBS interlayer in 1× PBS solutions[1]. The inserted schematic shows the measurement setup of EIS. **b**, Nyquist plot from the measured result, and fitting curve using Randles model (equivalent circuit inserted) is also plotted.

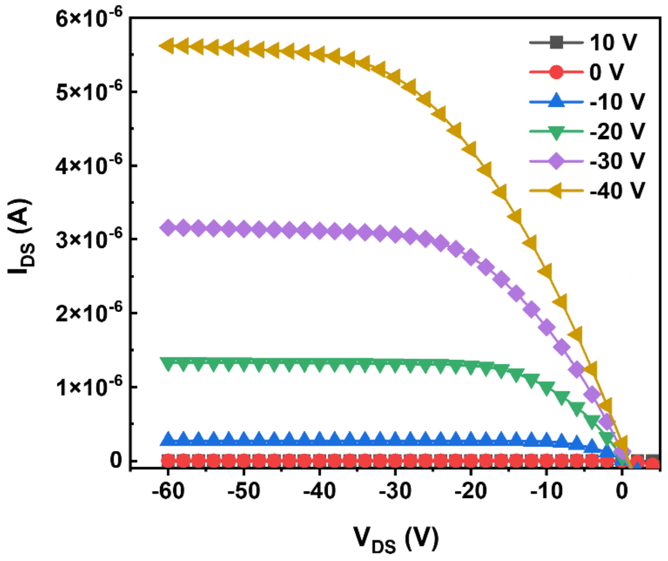

Supplementary Fig. 14. Output curves of the ultrasoft transistor device with different gate voltages from 10 V to -40 V (each step, 10 V).

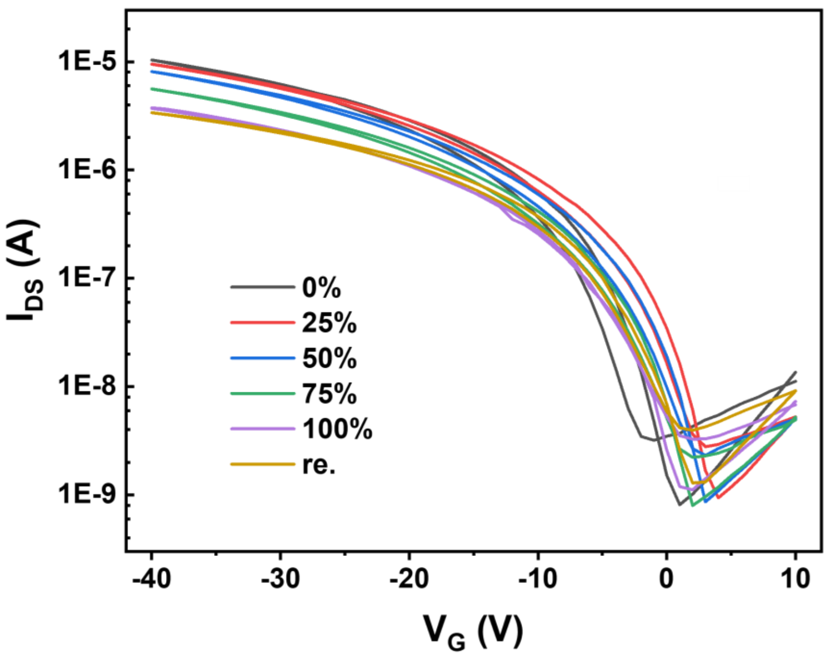

Supplementary Fig. 15. Transfer curve of the ultrasoft transistor device during stretching (0% to 100% and release state). V_{DS} = -60 V, V_{GS} = 10 to -40 V.

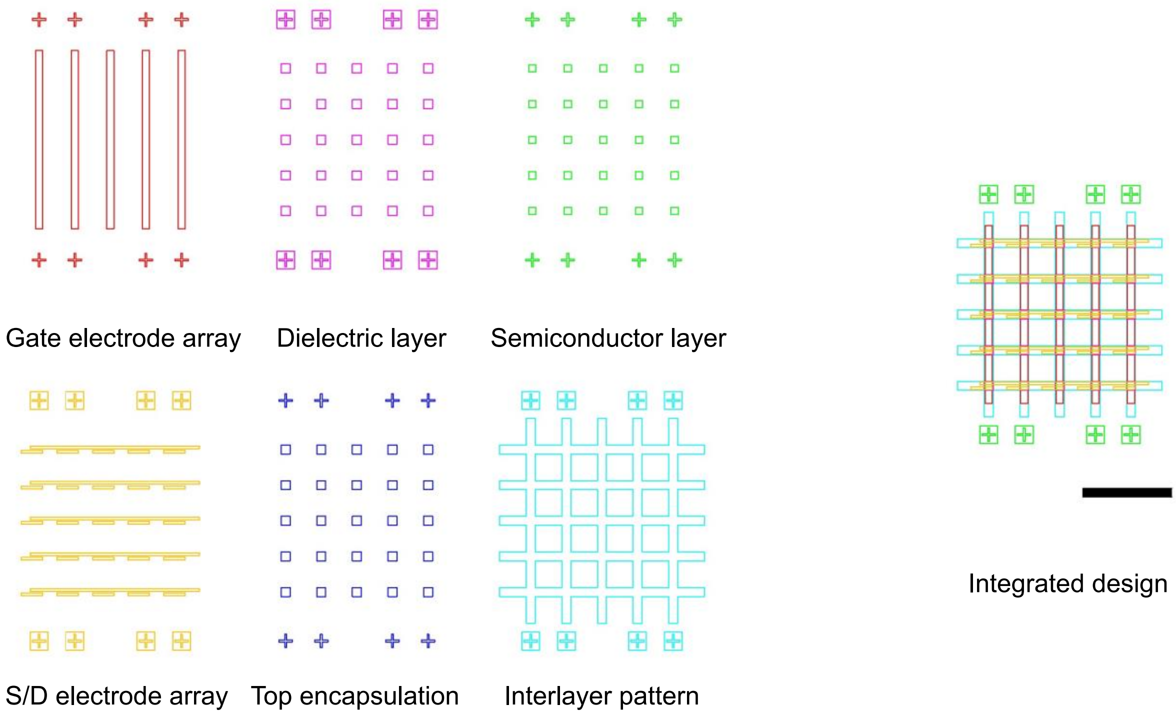

Supplementary Fig. 16. Layout of each layer in the active array. Transistors in the active array have a channel length of 200 μm and a channel width of 1 mm. The distance between each transistor unit is 3 mm in the horizontal direction and 3 mm in the vertical direction. The patterning of the interlayer fully covers all the transistors and the interconnections. Thicknesses of different layers are same to those in the single transistor design (scale bar, 10 mm).

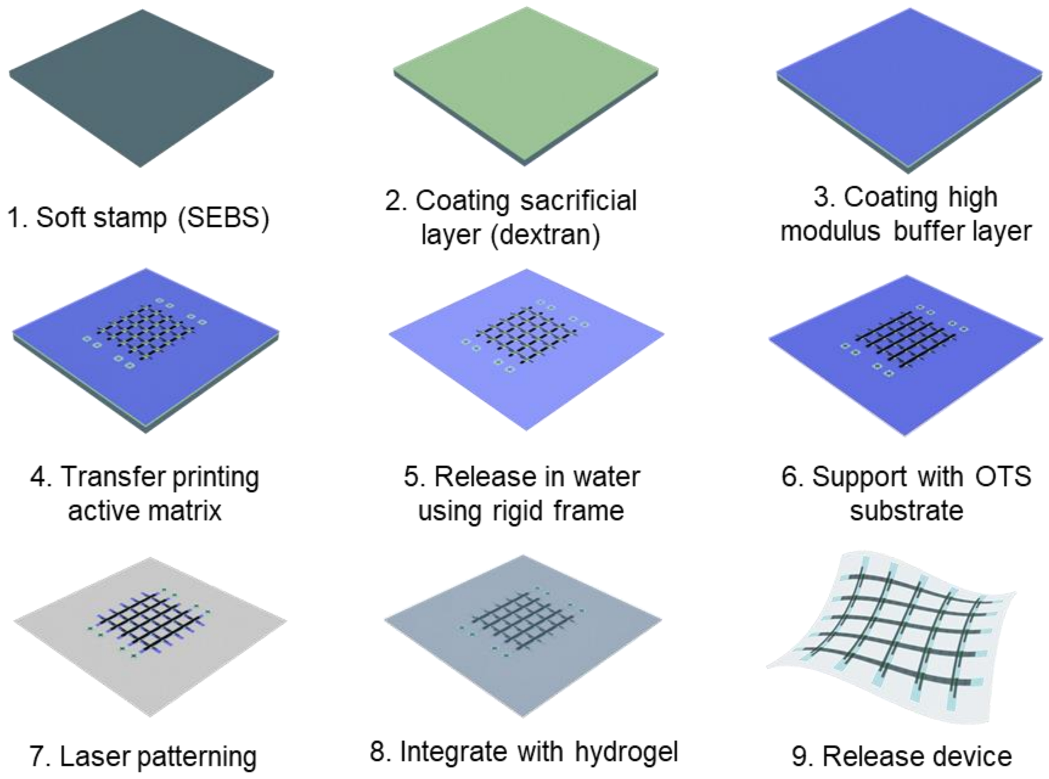

Supplementary Fig. 17. Fabrication process of the ultrasoft active matrix.

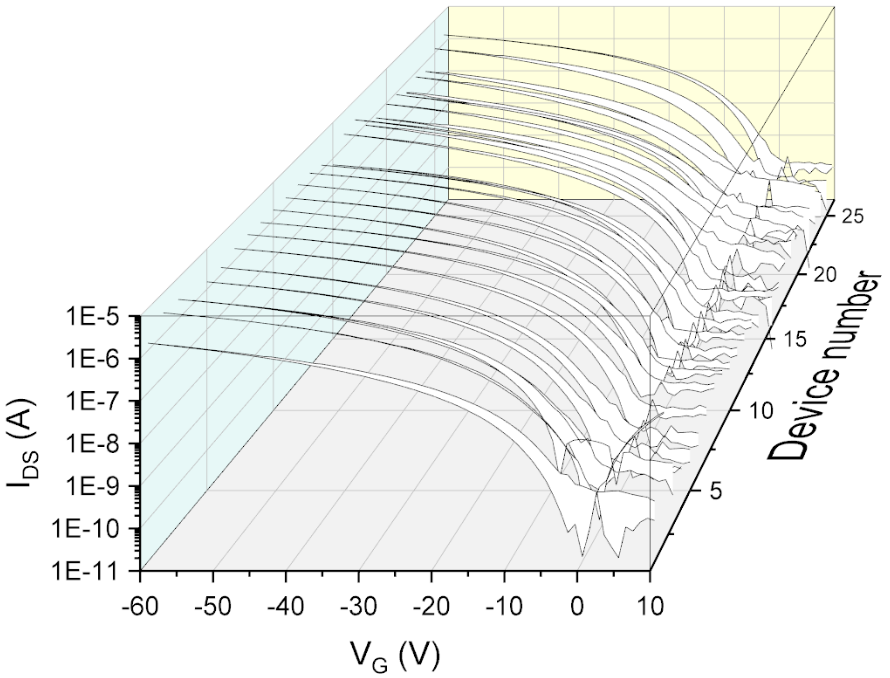

Supplementary Fig. 18. Transfer curves of individual transistor devices in the ultrasoft active matrix.

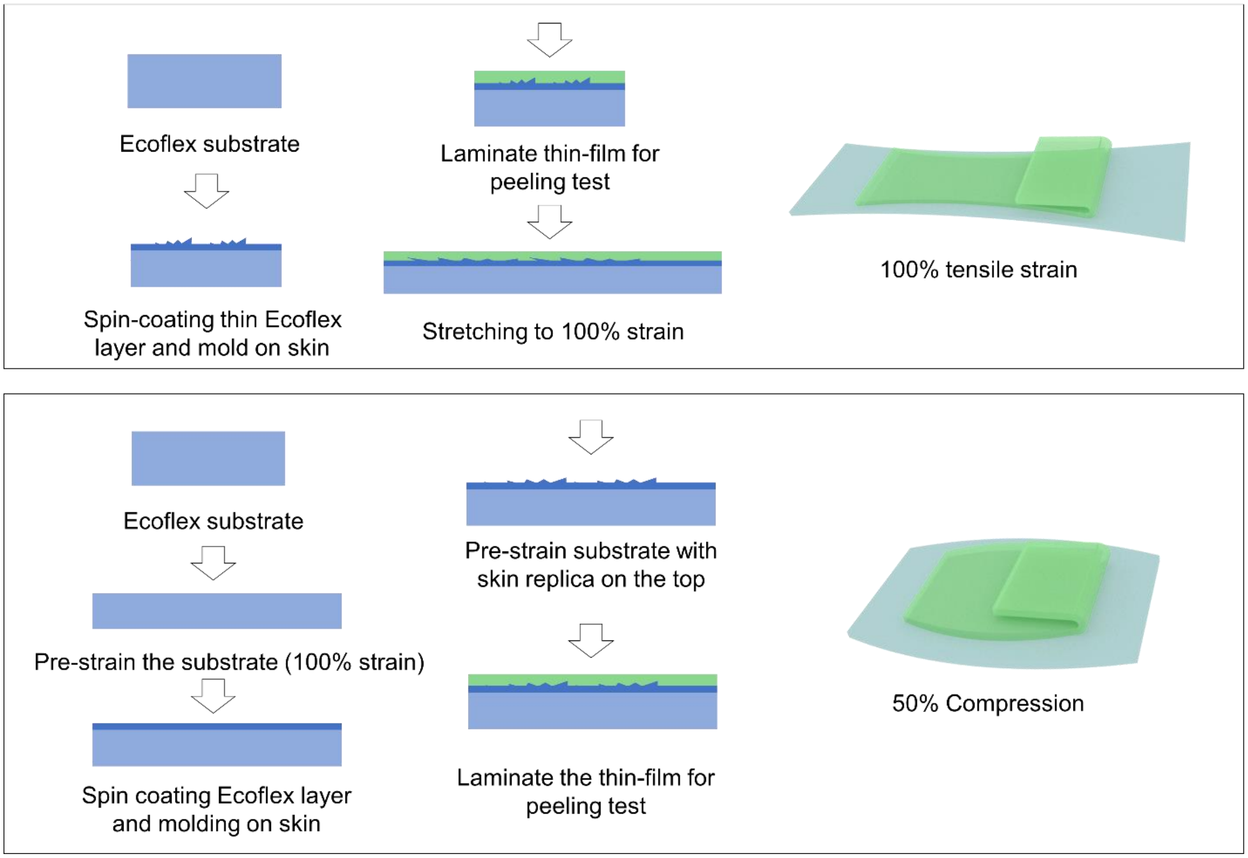

Supplementary Fig. 19. Sample preparation for the peeling test on a skin replica under different deformation conditions. The top panel is the preparation process of the sample with 100% tensile strain. The bottom panel is the preparation process for the peeling test on a 50% compressed skin replica.

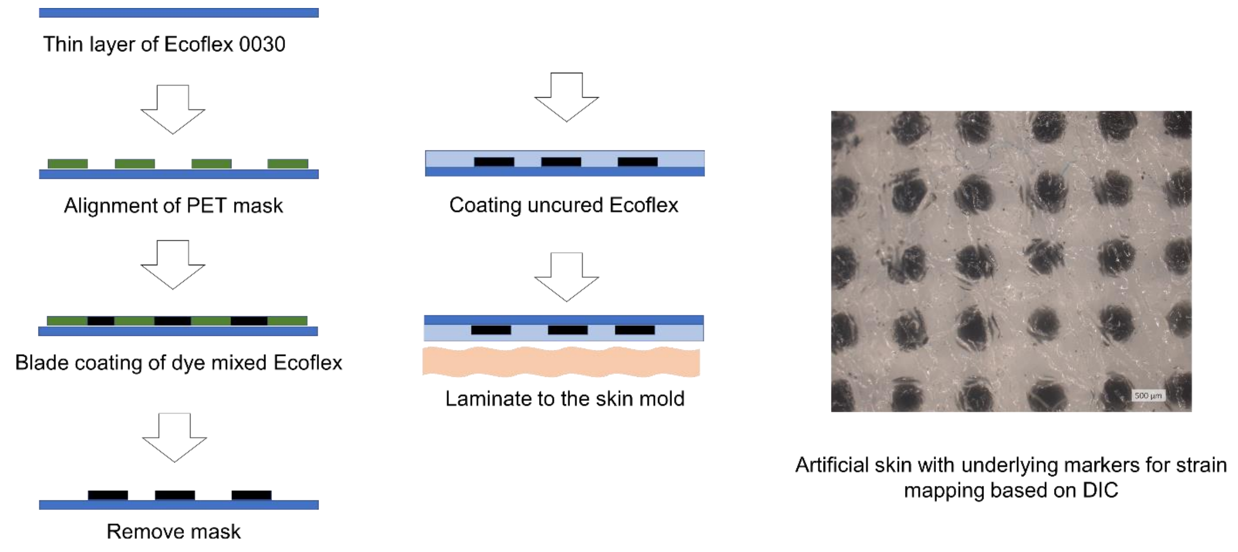

Supplementary Fig. 20. Fabrication process of a skin replica with strain mapping markers based on Ecoflex-0010. The black dots help to provide a high resolution for the DIC process. The diameter of the dot is 500 μm, and the interval between the two dots is 1 mm.

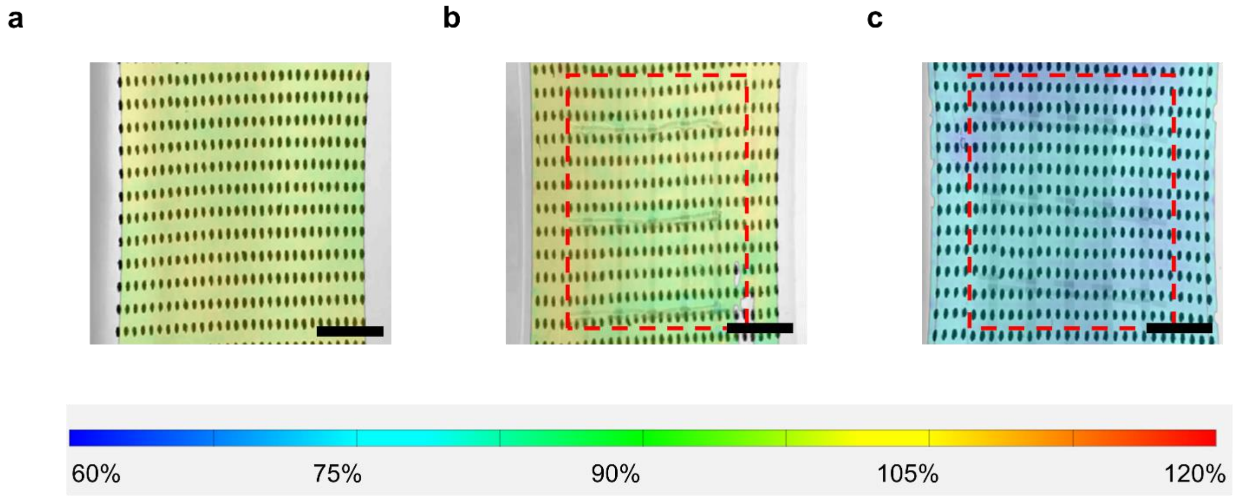

Supplementary Fig. 21. DIC analysis for skin replicas with ultrasoft or conventional substrates based active transistor-array device attached on top. a, DIC-based strain mapping of a skin replica that is without any device attached (stretched to 100% strain). **b**, Strain mapping of an ultrasoft device integrated with the skin replica (100% strain). **c**, A conventional device integrated with skin replica under 100% strain. The device areas are labeled with the red dashed line. No excessive mechanical constraint is introduced by the ultrasoft stretchable device. However, the conventional device shows strong mechanical constraints to the skin replica, which limits the deformation in the device-covered area (scale bars, 5 mm).

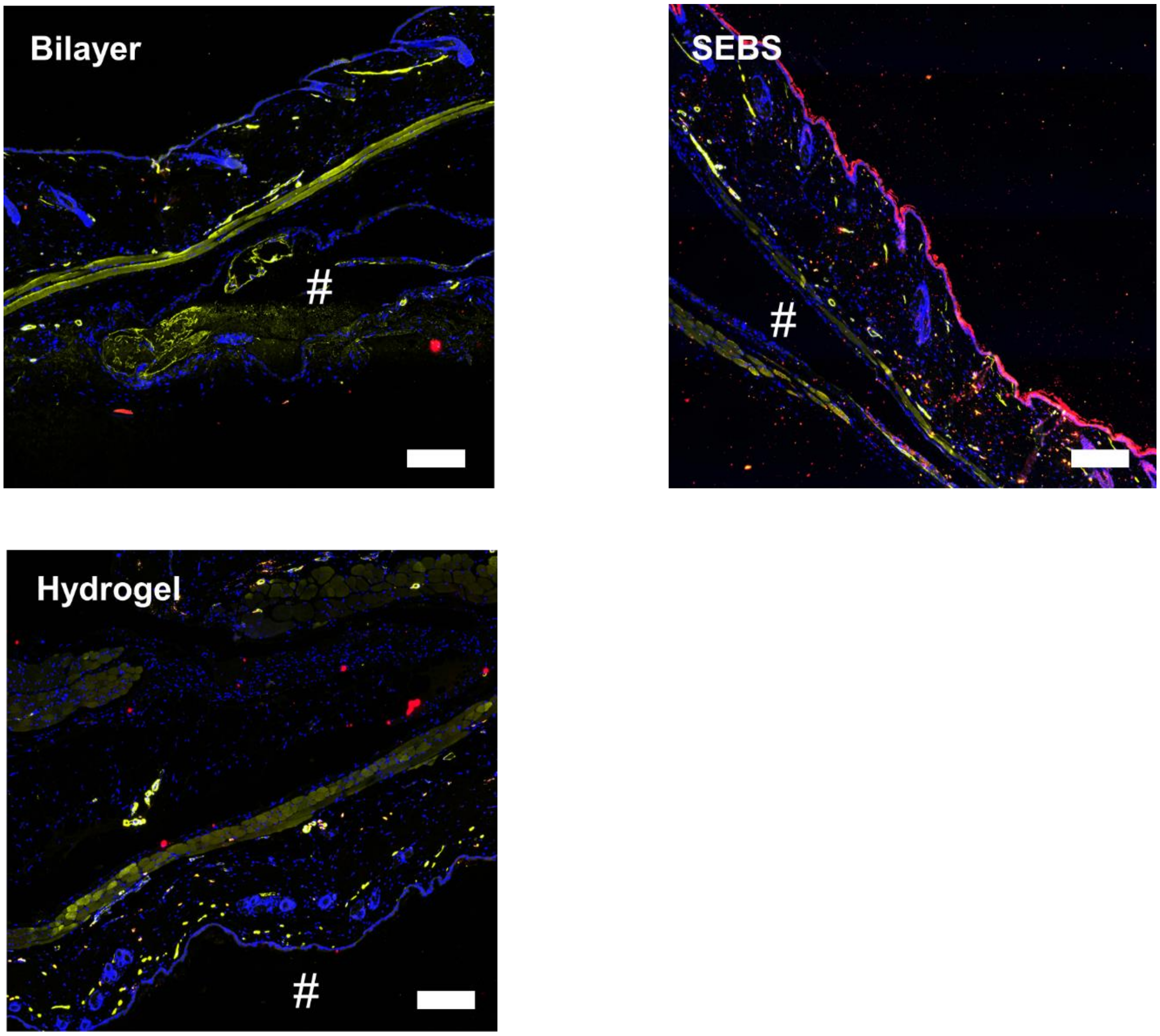

Supplementary Fig. 22. Representative fluorescence study for the immune response. α-SMA and CD68 are used for the analysis. α-SMA is for the collagen, and CD68 is for the macrophages. Higher intensity of α-SMA and CD68 are found around the SEBS implants than the bilayer and hydrogel implants (scale bar, 200 μm).

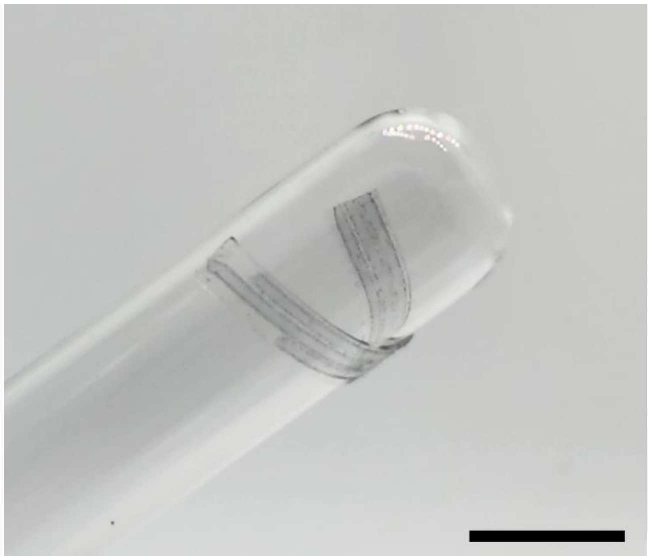

Supplementary Fig. 23. PEDOT:PSS composite based EP electrodes warp on the bottom of a test tube, which shows its conformability. Exposed sensing sites are 0.8 mm by 0.8 mm (scale bar, 10 mm).

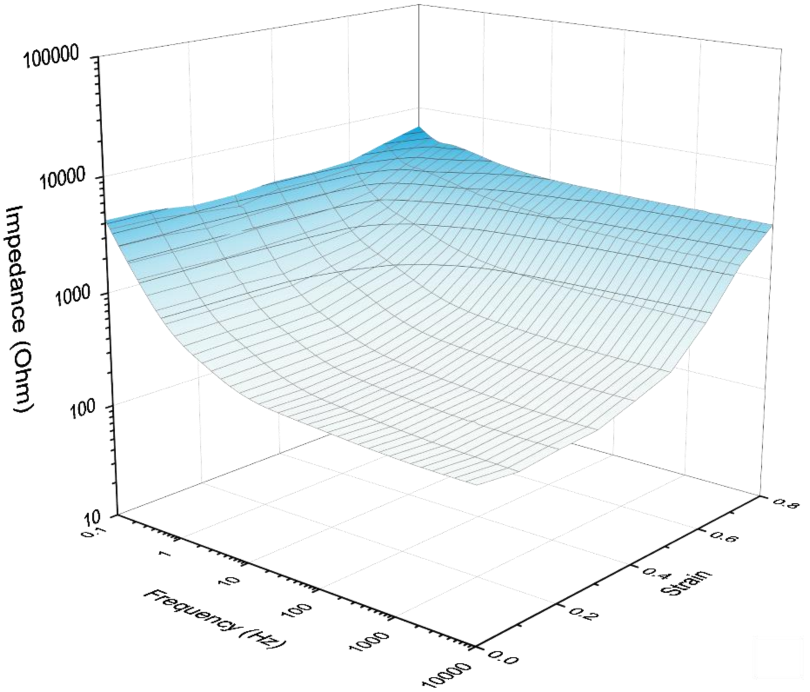

Supplementary Fig. 24. Impedance measurement for the PEDOT:PSS composite based EP electrodes under different strain levels.

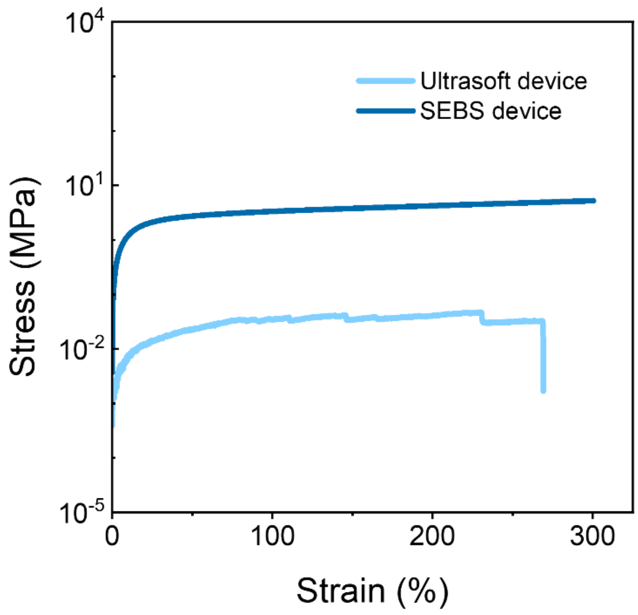

Supplementary Fig. 25. Modulus measurement for the EP electrode array based on the ultrasoft interlayer design and a conventional SEBS substrate.

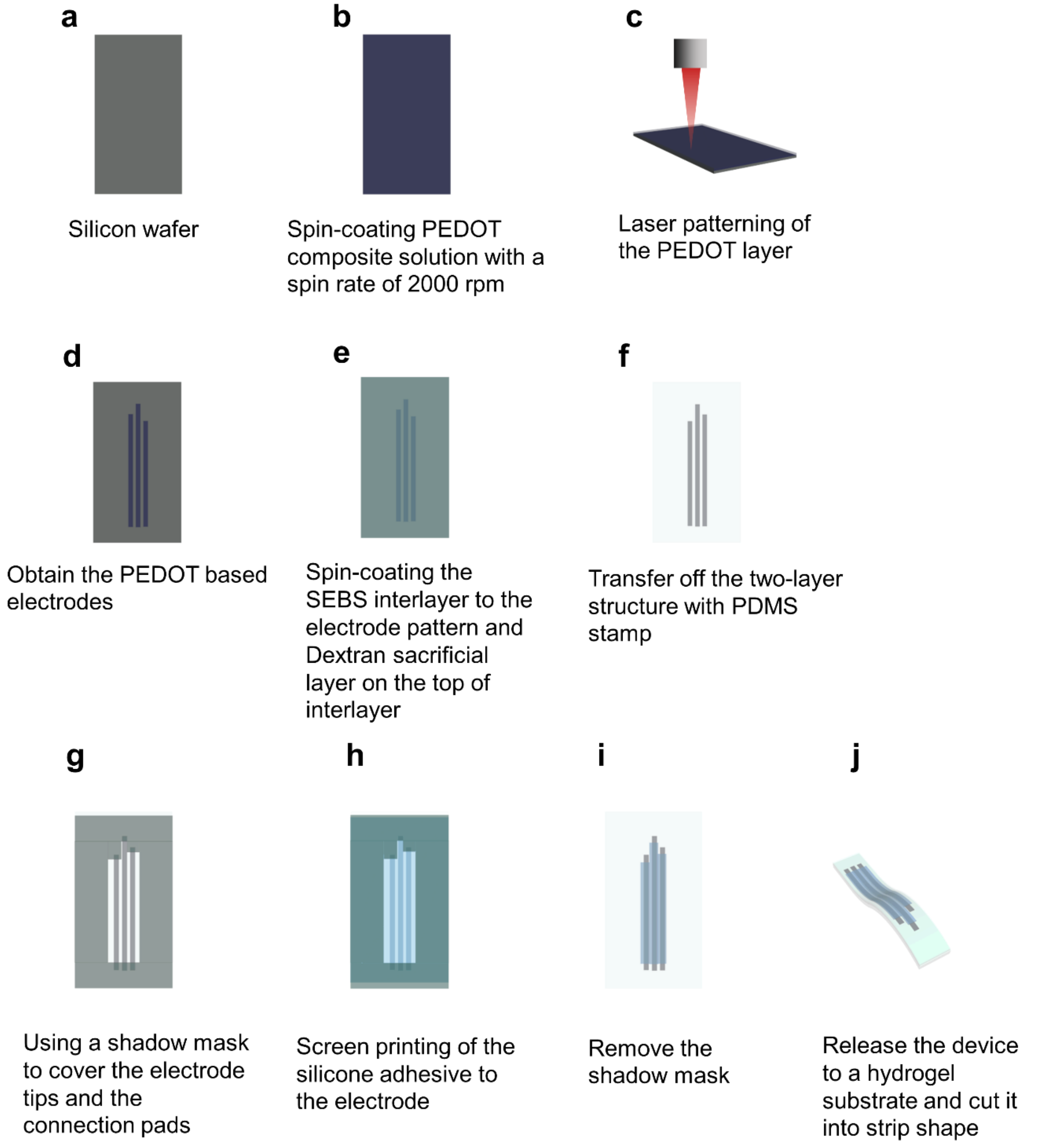

Supplementary Fig. 26. The fabrication process of the ultrasoft EP device. a-d, spin-coating and laser patterning of the PEDOT: PSS composite. **e-h,** building encapsulation and connections for the EP sensor. **i-j,** integrating electrodes with hydrogel substrate.

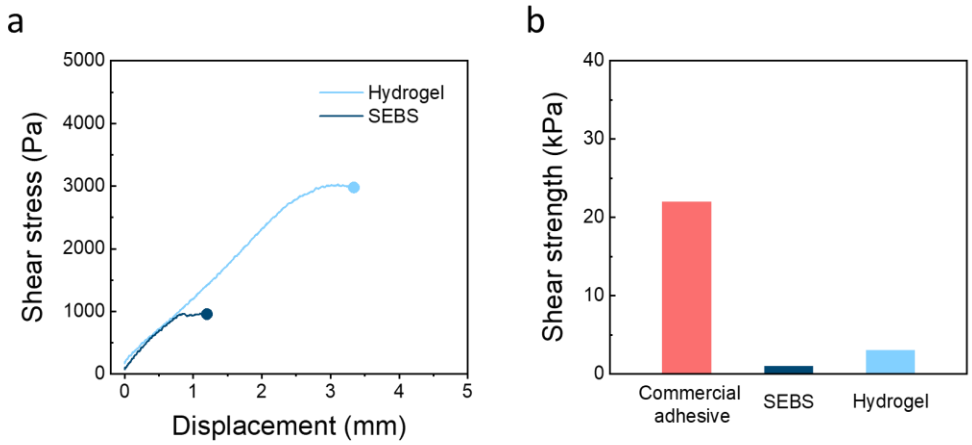

Supplementary Fig. 27. a, shear strength of the hydrogel film and SEBS film on heart tissue slice[2]. b, Shear strength of hydrogel, SEBS, and a commercial adhesive (with the value extracted from ref. 2).

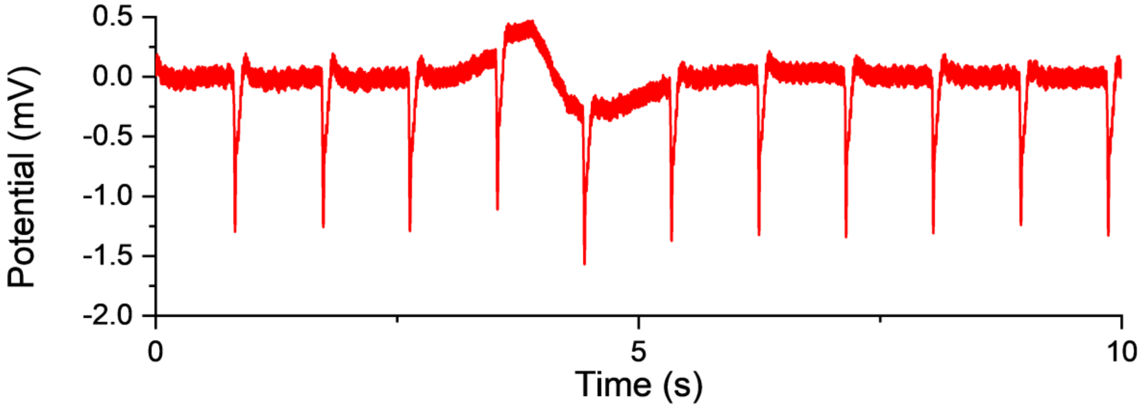

Supplementary Fig. 28. Typical ECG signal recorded with the ultrasoft electrodes. The calculated signal to noise ratio is 19.65 dB, using the equation, SNR=20 $\log_{10}(V_{\mathrm{signal}}/V_{\mathrm{noise}})$.

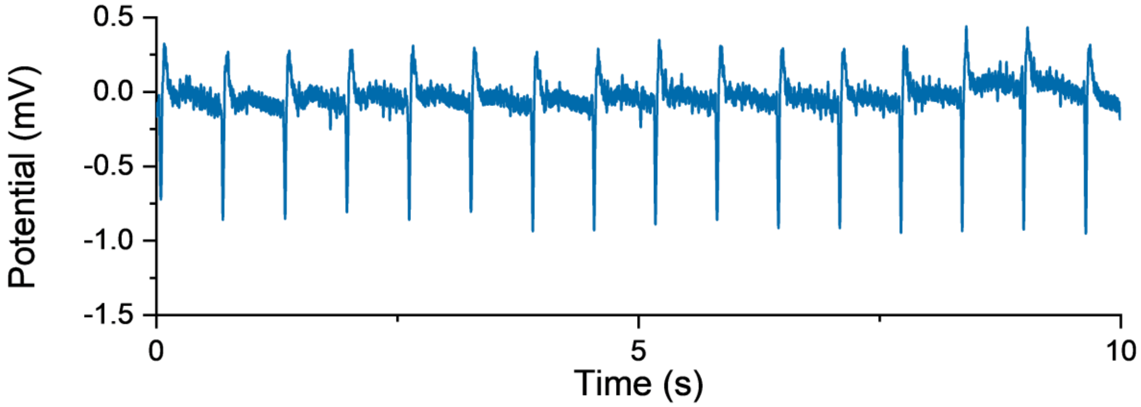

Supplementary Fig. 29. Typical ECG signal recorded with the SEBS-based electrodes. The calculated signal to noise ratio is 16.73 dB, using the equation, SNR=20 $\log_{10}(V_{signal}/V_{noise})$.

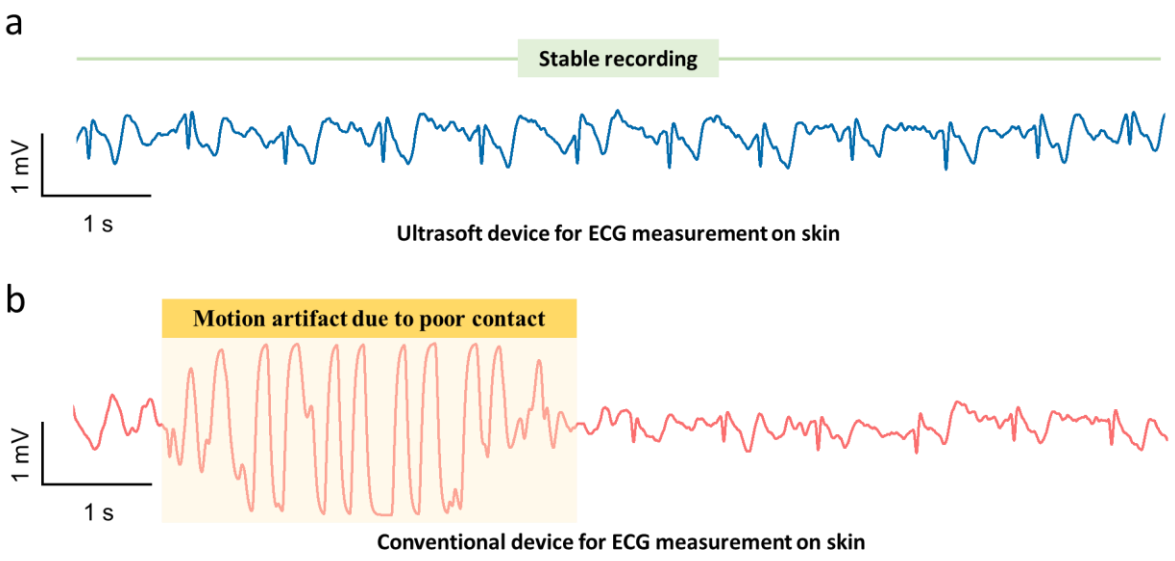

Supplementary Fig. 30. Stretchable PEDOT: PSS electrodes for ECG recording on skin surface. a, electrodes based on SEBS interlayer and ultrasoft hydrogel substrates. The low effective modulus enables good contact with skin and remains stable for the ECG recording. **b,** stretchable electrode based on SEBS substrate is less conformal and results in recorded signal with lower amplitude. Also, the high stiffness makes it hard to remain intimate contact with skin when the arm is moving, which further results in a distorted signal.

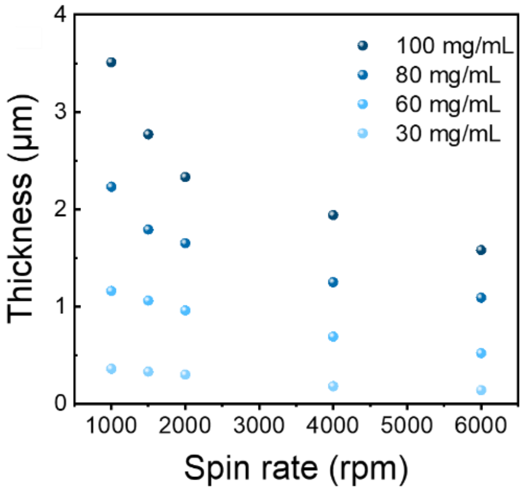

Supplementary Fig. 31. Relationship between SEBS interlayer thickness and spin-coating rate. Four concentrations are used to generate SEBS interlayers ranging from 140 nm to 3.5 µm

Supplementary Table 1. Details of interlayer design for electrically functional materials[3,4].

Functional materials (interlayer materials)	Modulus mismatch	Interfacial toughness	Optimized thickness	Stretchability
PEDOT: PSS & PFI (SEBS)	1.05 GPa (2.83 MPa)	14.2 J·m^{-2}	>10 μm	>80%
DPPT-TT & SEBS (SEBS)	1.94 MPa (2.83 MPa)	402.7 J·m^{-2}	>1 μm	>100%
CNT (SEBS)	4.1 GPa (2.83 MPa)	178.8 J·m^{-2}	>0.5 μm	>200%
AgNWs (TPU)	0.88 GPa (9.6 MPa)	269.7 J·m^{-2}	>0.5 μm	>180%

Supplementary Table 2. Details of interlayer design for electrically functional materials.

Key design feature	Stretchability	Substrate modulus	Device type	Reference
Au coated polyurethane nanomesh	30%	0.274 MPa	Passive	10
PEDOT: PSS hydrogel	20%	30 kPa	Passive	28
PAAm-PEDOT: PSS hydrogel	N.A.	2.5-4.0 GPa (polyimide)	Passive	33
Ag flakes in PAAm-alginate hydrogel	250%	3-4 kPa	Passive	35
DPPT-TT & SEBS composite	100%	1 MPa	Active	36
N2200 with PU encapsulation	50%	0.8~2.5 MPa (PDMS)	Active	37
P3HT-NFs & PDMS composite	30%	0.7 MPa	Active	49
Soft interlayer	100%	5.2 kPa	Active	Our work

Supplementary References

1. Floch, P. L. *et al.* Fundamental Limits to the Electrochemical Impedance Stability of Dielectric Elastomers in Bioelectronics. *Nano Lett* **20**, 224–233 (2019).
2. Yuk, H. *et al.* Dry double-sided tape for adhesion of wet tissues and devices. *Nature* **575**, 169–174 (2019).
3. Li, X., Gao, H., Murphy, C. J. & Caswell, K. K. Nanoindentation of Silver Nanowires. *Nano Lett.* **3**, 1495–1498 (2003).
4. Chen, L. *et al.* Auxetic materials with large negative Poisson's ratios based on highly oriented carbon nanotube structures. *Appl. Phys. Lett.* **94**, 253111 (2009).
