# Supplementary material for: Achieving tissue-level softness on stretchable electronics through a generalizable soft interlayer design
Source: Nat Commun. 2023 Jul 26;14:4488. doi: 10.1038/s41467-023-40191-3 (PMC10372055; doi:10.1038/s41467-023-40191-3)
Supplement: Supplementary file 3 — Description of Additional Supplementary Files [file 41467_2023_40191_MOESM3_ESM.pdf]

Description of Additional Supplementary Files

File Name: Supplementary Movie 1
Description: An ultrasoft device on an isolated heart
An ultrasoft electrode array was attached to a beating mouse heart for ECG measurement, and no obvious movement of the device was found during the ECG recording. The video was recorded for 6 minutes and 40 seconds (accelerated by 16 times).

File Name: Supplementary Movie 2
Description: A conventional device on an isolated heart
A conventional device based on SEBS substrate was attached to a beating mouse heart for ECG measurement. The electrodes shifted up and rotated to the right within 1 minute during the recording. The video was recorded for 64 seconds (accelerated by 4 times).
